# Quantitative structure-activation barrier relationship modeling for Diels-Alder ligations utilizing quantum chemical structural descriptors

**DOI:** 10.1186/1752-153X-7-171

**Published:** 2013-10-30

**Authors:** Sisir Nandi, Alessandro Monesi, Viktor Drgan, Franci Merzel, Marjana Novič

**Affiliations:** 1 Laboratory of Chemometrics, National Institute of Chemistry, Hajdrihova 19, Ljubljana 1000, Slovenia; 2 Laboratory of Biomolecular Structure, National Institute of Chemistry, Hajdrihova 19, Ljubljana 1000, Slovenia

**Keywords:** Quantitative structure-activation barrier relationships, Quantum chemical structural descriptors, HOMO LUMO Energy, Diels-Alder ligations, Multivariate linear regression and artificial neural network modelling

## Abstract

**Background:**

In the present study, we show the correlation of quantum chemical structural descriptors with the activation barriers of the Diels-Alder ligations. A set of 72 non-catalysed Diels-Alder reactions were subjected to quantitative structure-activation barrier relationship (QSABR) under the framework of theoretical quantum chemical descriptors calculated solely from the structures of diene and dienophile reactants. Experimental activation barrier data were obtained from literature. Descriptors were computed using Hartree-Fock theory using 6-31G(d) basis set as implemented in Gaussian 09 software.

**Results:**

Variable selection and model development were carried out by stepwise multiple linear regression methodology. Predictive performance of the quantitative structure-activation barrier relationship (QSABR) model was assessed by training and test set concept and by calculating leave-one-out cross-validated Q^2^ and predictive R^2^ values. The QSABR model can explain and predict 86.5% and 80% of the variances, respectively, in the activation energy barrier training data. Alternatively, a neural network model based on back propagation of errors was developed to assess the nonlinearity of the sought correlations between theoretical descriptors and experimental reaction barriers.

**Conclusions:**

A reasonable predictability for the activation barrier of the test set reactions was obtained, which enabled an exploration and interpretation of the significant variables responsible for Diels-Alder interaction between dienes and dienophiles. Thus, studies in the direction of QSABR modelling that provide efficient and fast prediction of activation barriers of the Diels-Alder reactions turn out to be a meaningful alternative to transition state theory based computation.

## Background

Activation energy is the minimal energy required to start a chemical reaction. It corresponds to the potential barrier separating the minima of potential energy of reactants and products. The free energy of activation is reversible work required to bring the system from the minimum of reactive well to the top of the barrier. For basically all the condensed phase reactions the transition state is valid and in order to calculated the rate constant it is enough to calculate the activation free energy. Still locating the transition state is not easy and therefore one has to proceed with computationally less demanding methods. The free energy difference between initial geometry-optimized reactants and the transition state at maximal potential energy barrier is correlated with the rate constant or reactivity of the reactant molecules. Structure-reactivity relationship is a variant of structure–property-activity relationship studies [1,2]. The concept of quantitative structure–property-activity relationship (QSAR/ QSPR) was originated from the idea of Crum-Brown and Fraser as early as 1870 when they proposed that biological response was a function of chemical structure. Although studies in structure–property-activity relationships go back to antiquity since the times of Crum-Brown and Fraser [3], it is only in the recent times that one witnesses a vigorous study of it as an interdisciplinary area of molecular design and modelling by developing quantitative relationship between molecular activity or property (such as partition coefficient (log *P*), boiling point, melting point, acid and base constant, chromatographic retention index, toxicity, or reactivity) and theoretical structural properties such as constitutional, electrostatic, geometrical, topological, or quantum chemical molecular characteristics [4-8]. A significant dimension to such studies has been extended largely due to the approach from the view point of developing quantitative relationship between reactivity and theoretical structural quantum chemical properties by soft computations which increase the probability of success and reduce the time and cost involvement in the chemical design, discovery and modelling of potential reactions and candidates [9-11]. But there are only a few quantitative structure-activation barrier relationship studies using quantum chemical structural parameters concerning the modelling of promising chemical reactions [12,13].

One of the important fields of bio orthogonal chemistry for designing of efficient reactions is well known Diels-Alder ligation because of its higher rate and selectivity in water [14-21]. The Diels–Alder reaction is an organic cycloaddition between a conjugated diene and a substituted alkene, commonly termed the dienophile, to form a substituted cyclohexene system. Otto Paul Hermann Diels and Kurt Alder first documented the novel reaction in 1928 for which they were awarded the Nobel Prize in Chemistry in 1950 for their work on the eponymous reaction. The Diels–Alder reaction is generally considered as one of the more useful reactions in organic chemistry since it requires very little energy to create a cyclohexene ring, which is useful in many other organic reactions [22-26] to gain insights into structure, dynamics and function of bio molecules. Diels-Alder ligation of electron-rich hexadiene and electron-deficient maleimide has been found to be facilitated by electron withdrawing groups (e.g., C = O and C ≡ N) on the dienophile and electron-donating groups (e.g., –R and –OR) on the diene which would be effective for the bioconjugation of peptides [27], small molecules [28,29], and oligonucleotides [30,31]. It has also been used for the bio-conjugation of carbohydrate to proteins [32,33].

There have been a number of successful explanations regarding the Diels–Alder reactions from the view point of molecular orbital theory. Woodward, Hoffmann, and Fukui used molecular orbital theory to explain that overlapping between *p* orbitals of the substituents on the dienophile with *p* orbitals of the substituents on the diene is favourable, helping to bring the two molecules together [34-36]. Tang et al. [13] carried out a systematic theoretical study based on M06-2X/6-31 + G(d)//B3LYP/6-31G(d) level on the design of new dienophiles in order to extend the scope of Diels–Alder ligation. A drawback of the Diels–Alder ligation is that the widely used maleimide moiety as a typical Michael acceptor can readily undergo Michael addition with nucleophiles in living systems. Thus, Tang et al. calibrated a theoretical method to calculate the activation barriers of Diels–Alder reactions by benchmarking the calculations against the available experimental data for 72 non-catalysed Diels-Alder ligations. They have also calculated Diels-Alder barriers of σ-electron withdrawing group substituted alkenes, cyclic alkenes with consideration of electronic and ring strain effect and barriers of Diels-Alder and thiol addition reactions of designed alkenes which are efficient reactions and nucleophile-tolerant in living system. The method is time consuming and due to its complexity sometimes it fails to optimize the reactant complex at a transition state level.

Due to the above reasons, an attempt has been made in the present investigation to find an alternative and cheaper theoretical method to evaluate activation barriers of the Diels-Alder reactions based on quantitative structure-activation barrier relationship (QSABR) modelling utilizing theoretical quantum chemical descriptors calculated solely from the chemical structure of the ligation reactant molecules. The energies of the highest occupied molecular orbital (HOMO) and the lowest unoccupied molecular orbitals (LUMO) are quantum chemical quantities that can govern the chemical reactions. They are calculated from the structures of reactant molecules utilizing quantum-chemical methods, which can explain reactivity correlated with the activation barriers of a complete molecule as well as of molecular fragments and substituents [37,38]. Computed descriptor based QSABR model produces comparable results as those calculated by Tang et al. at more complicated transition state theory based calculation using M06-2X/6-31 + G(d)//B3LYP/6-31G(d). The QSABR model was validated by introducing training and test set concept and was then applied for the prediction of Diels-Alder barriers of alkenes substituted with σ-electron withdrawing groups, cyclic alkenes and cyclopropene derivatives. The present protocol based on computed quantum chemical descriptors based on HOMO and LUMO energies of reactants can successfully predict activation barriers of σ-electron-withdrawing-group-substituted cyclopropenes, cyclic alkenes and barriers of Diels-Alder reactions studied by Tang et al. [13] at a more computationally demanding and not always successful transition state level. The proposed modelling methodology can be a useful tool to obtain the structure-activation barrier relationships of bio molecules and thus propose new ligations in click chemistry. The computational approach developed is a potential theoretical bench mark for the design of efficient and selective Diels-Alder ligation reactions.

## Results and discussion

### Computation of quantum chemical descriptors

We have calculated 24 quantum chemical properties using HOMO and LUMO energetics of the dienes and dienophiles involved in 72 reactions considered in this study. The activation barriers calculated using the Eyring–Polanyi equation [2] with experimental reaction rates obtained from literature [19,39-43] have been taken into account to formulate quantitative structure-activation barrier relationships modelling. Quantum chemical structural descriptors were calculated from structures of standalone reactants, different dienes and dienophiles listed in Additional file 1: Table S1. Some of the reactions were chosen for the test set; see labelling of ID numbers of reactions, entries of the first column, and the explanation in the footnote to Additional file 1: Table S1. Chemical structures of dienes and dienophiles along with activation barrier data for all reactions are given in Additional file 1: Table S1.

The calculated descriptors are briefly described in Table 1. Hardness (*η*), softness (*SOF*), electronegativity (*χ*), electrophilicity (*ω*), and dipole moment, which are calculated by Gaussian09 [44], and orbital interaction energy difference (*∆E*), are the important electronic structure features used to describe stability, reactivity, chemical potential and other related properties of molecules, see Eqs. (1, 2, 3, 4 and 5). Hardness has been used to understand chemical reactivity and stability of molecules. Electronegativity was introduced by Pauling as a power of an atom in a molecule to attract electron to itself. Softness is a property of molecule that measures the extent of chemical reactivity and is obtained as reciprocal value of hardness. Electrophilicity was proposed as a measure of energy lowering due to maximal electron flow between donor and acceptor. The most obvious and most often used quantity to describe the polarity is the dipole moment of the molecule. The total dipole moment, however, reflects only the global polarity of a molecule.

**Table 1:** List of calculated quantum chemical properties used in this study

**No.**	**Notation**	**Definition**
1	HOMO_d_	Highest occupied molecular orbital energy of the diene
2	LUMO_a_	Lowest unoccupied molecular orbital energy of the dienophile
3	HOMO_a_	Highest occupied molecular orbital energy of the dienophile
4	LUMO_d_	Lowest unoccupied molecular orbital energy of the diene
5	(HOMO_d_ - LUMO_a_)	Difference between HOMO_d_ and LUMO_a_
6	(HOMO_a_ - LUMO_d_)	Difference between HOMO_a_ and LUMO_d_
7	∆*E*	Orbital interaction energy difference between the corresponding orbitals of the reactants as calculated by the formula proposed by Sustmann.
8	*η*_d_	Hardness of the diene
9	*η*_a_	Hardness of the dienophile
10	(*η*_d_ - *η*_a_)	Difference between Hardness of the diene and dienophile
11	*χ*_d_	Electronegativity of the diene
12	*χ*_a_	Electronegativity of the dienophile
13	(*χ*_d_ - *χ*_a_)	Difference between electronegativity of the diene and dienophile
14	*ω*_d_	Electrophilicity of the diene
15	*ω*_a_	Electrophilicity of the dienophile
16	(*ω*_d_ - *ω*_a_)	Difference between electrophilicity of the diene and dienophile
17	∆HOMO	Difference between HOMO levels of the diene and dienophile
18	∆LUMO	Difference between LUMO levels of the diene and dienophile
19	SOF_d_	Softness of the diene
20	SOF_a_	Softness of the dienophile
21	∆SOF	Difference between softness of the diene and dienophile
22	DM_d_	Dipole moment of the diene
23	DM_a_	Dipole moment of the dienophile
24	∆DM	Difference between dipole moments of the diene and dienophile

^a^ dienophile; ^d^diene.

Inspired by the work of Maynard et al. [45], Parr and co-workers [46] have provided a definition of electrophilicity (*ω*) as

(1) $$\omega =\frac{\mu^{2}}{2\eta }=\frac{\chi^{2}}{2\eta }\;$$

where *μ* is the chemical potential, a Lagrange multiplier associated with the normalization of density, defined as

(2) $$\mu ={\left(\frac{\partial E}{\partial N}\right)}_{V\left(r\right)}=-\frac{I+A}{2}=\frac{E_{\mathrm{LUMO}}+E_{\mathrm{HOMO}}}{2}$$

*η* is the absolute hardness given by

(3) $$\mu ={\left(\frac{\partial^{2}E}{\partial N^{2}}\right)}_{V\left(r\right)}=\frac{I-A}{2}=\frac{E_{\mathrm{LUMO}}-E_{\mathrm{HOMO}}}{2}$$

and softness (*SOF*) is given by (1/*η*). It should be noted that softness, hardness and polarizability are related quantities as molecules with high hardness/low softness have low polarizability and vice versa. Moreover, Vela and Gazquez [47] demonstrated a linear relationship between polarizability and the global softness of a system.

Parr et. al. [46] define the electronegativity (*χ*) as the negative of chemical potential (-*μ*)

(4) $$\chi =-\mu ={\left(\frac{\partial E}{\partial N}\right)}_{V\left(r\right)}=\frac{I+A}{2}=-\frac{E_{\mathrm{LUMO}}+E_{\mathrm{HOMO}}}{2}$$

In the above definitions for an *N*-electron system with total energy *E* and external potential *V*(*r*), *I* and *A* are the ionization potential and the electron affinity, respectively, whereas *E*_HOMO_ and *E*_LUMO_ are the energies of the highest-occupied and lowest-unoccupied molecular orbitals, respectively. According to the Koopman’s theorem [48], *I* is simply the eigenvalue of HOMO with a negative sign and *A* is the eigenvalue of LUMO with a negative sign.

The interaction energy difference (*ΔE*) between the corresponding orbital of the dienes and dienophiles was calculated using the following formula proposed by Sustmann [49]:

(5) $$\Delta E=\frac{\left(\mathrm{HOM}O_{d}-\mathrm{LUM}O_{a}\right)+\left(\mathrm{HOM}O_{a}-\mathrm{LUM}O_{d}\right)}{\left(\mathrm{HOM}O_{d}-\mathrm{LUM}O_{a}\right)*\left(\mathrm{HOM}O_{a}-\mathrm{LUM}O_{d}\right)}$$

where *d* is referring to the dienes and *a* indicates dienophiles. In this simplified form of the interaction energy, we assume equal contributions of the atomic orbital coefficients at the centres where the new bonds are formed together with the resonance integral as a measure for the strength of the interaction of the reactants.

Quantum chemical descriptors have long been producing a crucial source of information for modelling chemical reactivity [50-56] and thus have great importance in the development of quantitative structure activation barrier relationships dealing with the chemical, physical, biochemical, and pharmacological properties of chemical compounds. A total number of 24 quantum chemical descriptors are calculated solely from the structures of dienes and dienophiles, which are subjected to model quantitative structure-reactivity and quantitative structure activation barrier relationships utilizing Stepwise-multiple linear regression methods.

### Linear QSABR model details

As it is shown in Additional file 1: Table S1, we have 72 Diels Alder reactions along with experimental activation barriers considered from the literatures. Stepwise-MLR method has been applied to develop quantitative structure activation barrier relationship modeling which focus influences of quantum chemical descriptors (described in Table 1) towards activation energy barriers that can be predicted easily with the application of such models avoiding complexity of the calculation at transition state level. QSABR model developed for all Diels-Alder reactions is given as

(6) $$\begin{array}{ll} \;\left(\mathrm{\Delta G}\right)= & -6.4\pm 8.5+\left(12.67\pm 3.4\right)\Delta SOF\\ & +\left(1846.00\pm 748\right)\omega_{d}-\left(148.00\pm 45\right)\Delta \text{HOMO}\\ & +\left(291.00\pm 73\right){\text{LUMO}}_{d}+\left(145.00\pm 54\right)\omega_{a}\\ & -\left(485.00\pm 252\right)\chi_{d} \end{array}$$

N = 72, R^2^ = 0.831, F = 53, Q_Loo_^2^ = 0.784, PRESS = 437.271, SE = 2.30

In the above equation, N, R^2^, Q_Loo_^2^, PRESS and SE represent number of observations, squared regression coefficient, leave-one-out (LOO) cross-validated R^2^, predictive sum of square deviations and standard error of the regression, respectively. These are commonly used measures of acceptance of QSAR models [57-59]. R^2^ and cross validated (LOO) Q^2^ of a model can be obtained from:

(7) $$R^{2}=1-\frac{\sum_{i=1}^{N}{\left[\Delta G_{\exp}-\Delta G_{\mathrm{calc}}\right]}^{2}}{\sum_{i=1}^{N}{\left[\Delta G_{\exp}-\bar{G}\right]}^{2}}\;$$

R^2^ is a measure of explained variance.

(8) $$Q_{\mathrm{Loo}}^{2}=1-\frac{\sum_{i=1}^{N}{\left[\Delta G_{\exp}-\Delta G_{\mathrm{pred}}\right]}^{2}}{\sum_{i=1}^{N}{\left[\Delta G_{\exp}-\bar{G}\right]}^{2}}\;$$

where, ∆G_exp_, ∆G_calc_, ∆G_pred_ indicate experimental, calculated and predicted activation barriers respectively and ∆̅G̅ indicates mean of experimental activation barrier values.

In this set of reactions, the computed quantum chemical structural descriptors represent a significant impact on the activation energy barriers. Eq. ([6]) can explain 83% and predict 78% of variance of the activation barriers of the studied reactions. LUMO level of the diene, difference between softness and difference between the HOMO levels of the dienes and dienophiles, electrophilicity of the dienes and dienophiles and electronegativity of the dienes can produce significant impact on the activation barriers.

Thus it is evident from this study that the application of quantum chemical structural properties can generate quantitative structure activation barrier relationship model with good prediction accuracy of the activation barriers. The model should be statistically validated prior to prediction of new reactions using such QSABR model.

### QSABR model validation and prediction of activation barriers

Further evaluation of model's stability and prediction ability has been performed by dividing the total data set (72 reactions) into training and test sets at a random basis, taking care of equal distribution of the objects over the whole reactivity space. Therefore both sets contain reactions from the available data space. Compounds with asterisk in Additional file 1: Table S1 were selected as test set comprising of 24% of the total reaction data. The quality of the training model is calculated by R^2^ and cross-validated Q_Loo_^2^ values for the training set and an external validation was performed by calculating predictive R^2^ (R_pred_^2^) for the test set reactions. Predictive R^2^ (R_pred_^2^) for the test set is calculated as

(9) $$R_{\mathrm{pred}}^{2}=1-\frac{\sum_{i=1}^{N}{\left[\Delta G_{\exp\left(\mathrm{test}\right)}-\Delta G_{\mathrm{pred}\left(\mathrm{test}\right)}\right]}^{2}}{\sum_{i=1}^{N}{\left[\Delta G_{\exp\left(\mathrm{test}\right)}-\bar{\Delta }{\bar{G}}_{\exp\left(\mathrm{train}\right)}\right]}^{2}}\;$$

where, ∆G_exp(test)_ and ∆G_pred(test)_ indicate experimental and predicted activation barriers of the test set and ∆̅G̅ _exp(training)_ indicates mean of experimental activation barriers of the training set. For a predictive model, the value of R_pred_^2^ should be more than 0.5 [60].

The quantitative structure activation barrier relationship model formulated by using stepwise-MLR method for the training reaction set is obtained as

(10) $$\begin{array}{ll} \;\Delta G= & -121.3\pm 43.7-\left(195.00\pm 49\right)\Delta \text{LUMO}\\ & +\left(1286\pm 333\right)\omega_{a}+\left(1127.00\pm 326\right)\left(\omega_{d}–\omega_{a}\right)+\\ & \left(493.00\pm 147\right){\mathrm{LUMO}}_{d}-\left(6.10\pm 2.5\right)\Delta E\\ & +\left(7.10\pm 3.9\right){SOF}_{d}+\left(0.21\pm 0.14\right){DM}_{a} \end{array}$$

N = 55, R^2^ = 0.865, F = 43, Q_Loo_^2^ = 0.800, PRESS = 300.461, SE = 2.06, R_pred_^2^ = 0.880.

This QSABR equation can explain and internally predict 86.5% and 80% of variances of the activation barriers of the studied training reactions and it can produce 88% of the external model predictability for the test reactions. Due to rather high error in coefficients shown in parentheses in Eq. ([10]), the applicability domain of the MLR model was assessed during the external validation procedure and reported below, in the chapter “Prediction of activation barriers for new reactions”. One can observe that many of the descriptors selected when using the whole data set are different from the descriptors in the model built using the training set only. The differences may occur because the descriptors used are correlated and the step-wise method can use one descriptor or a set of descriptors to replace another one while producing a model of similar quality. Due to a large search space several local minima of similar quality can be found, which all provide reasonably good predictions. Also the chemical properties represented in a selected set of descriptors and thus correlating most with the reaction barriers can be interpreted similarly.

Electrophilicity and dipole moment of the dienophile, electrophilicity differences between the reactants, LUMO level and softness of the dienes have positive impact on the activation barriers. It means that decreasing the values of these variables may produce lower activation barriers for the Diels-Alder reactions and may give higher rate of reactions. Negative coefficients of ∆*LUMO* and ∆*E* indicate that small increase in the difference between LUMO energies and orbital interaction energies of dienes and dienophiles may decrease activation barriers. This distinction is of fundamental importance from the physical chemist’s point of view and should be helpful for designing of promising Diels-Alder reactions prior to experimental synthesis.

**Figure 1:**
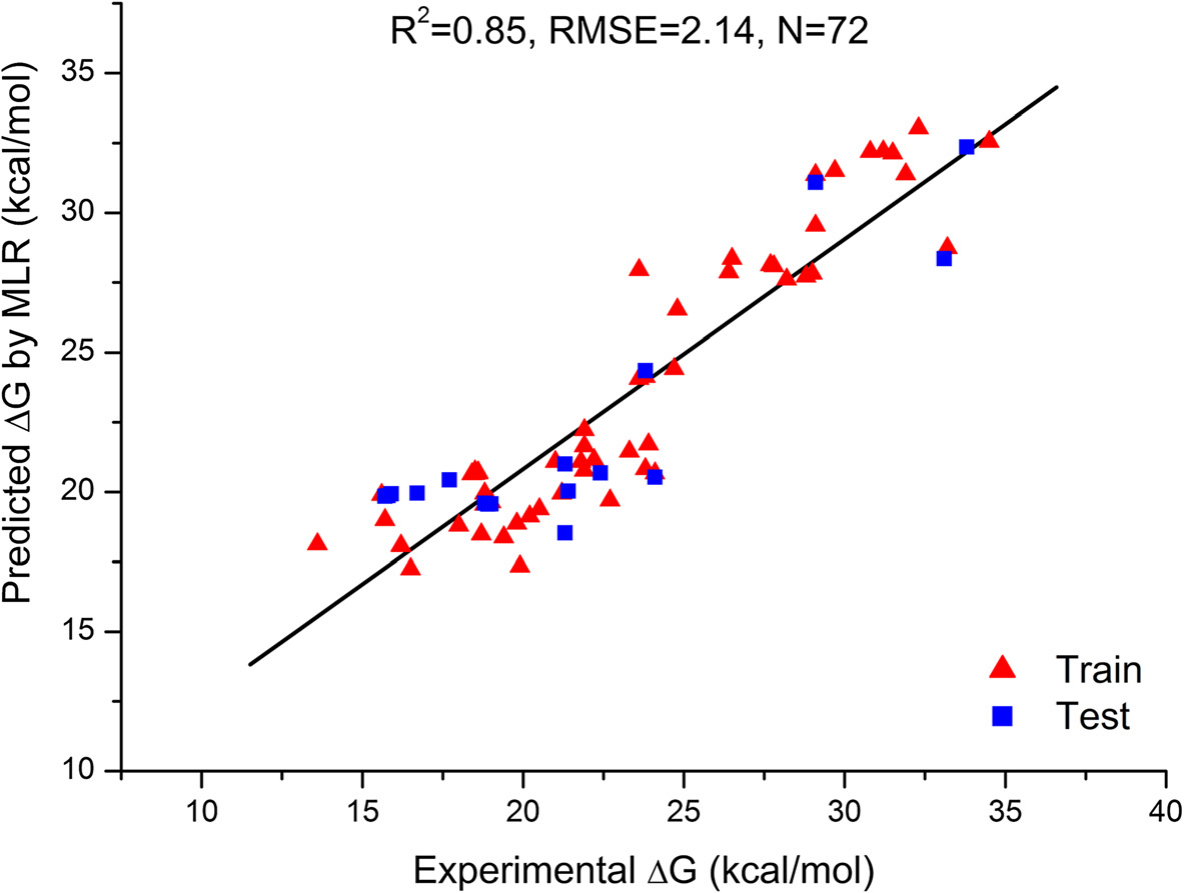
Plot of experimental and predicted activation barrier (∆G) for 72 reactions.

Eq. ([10]) has been used to predict activation barriers of the training and test reactions. Predicted activation barriers for all 72 observations are given in Additional file 1: Table S1. The plot of experimental versus predicted activation barriers for training and test reactions is represented in Figure 1. Root mean square error of prediction is 2.14 kcal/mol and linear correlation coefficient between experimental versus predicted activation barrier is R^2^ = 0.85 which is almost satisfying according to the linear correlation coefficient (R^2^ = 0.93) of the same reactions calculated by Tang et al. [13].

**Figure 2:**
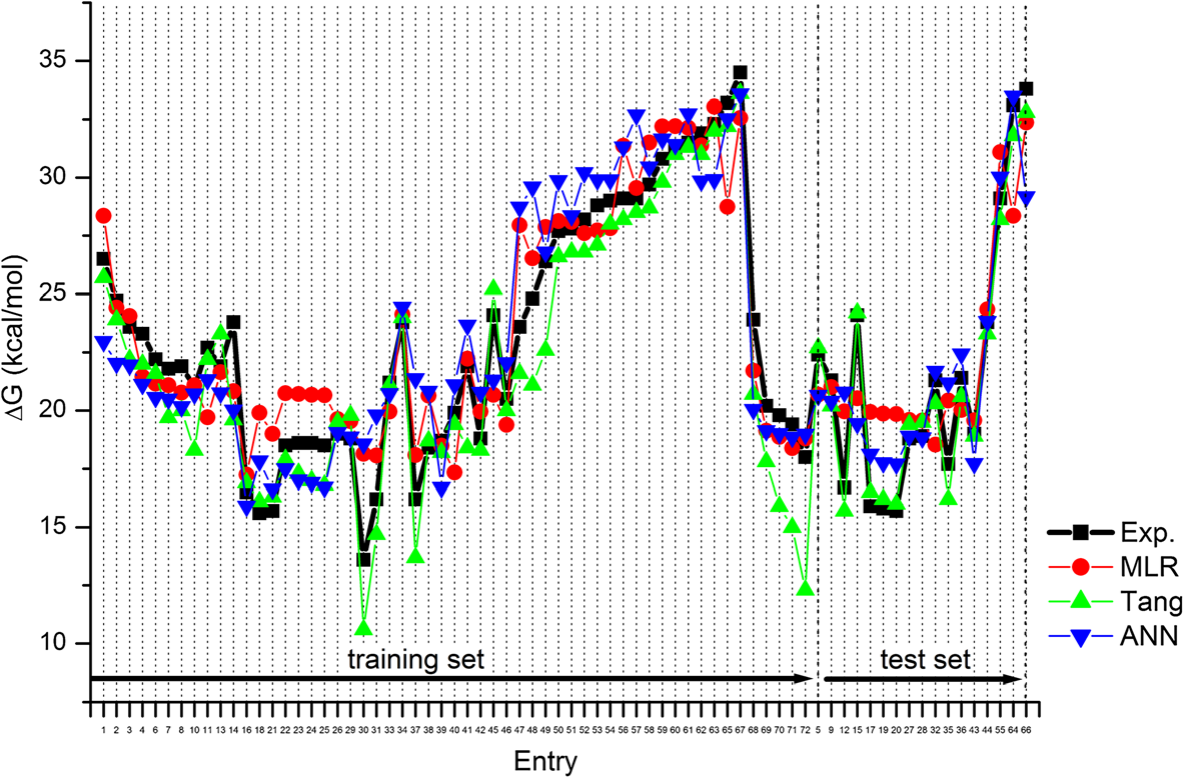
Comparison between experimental and predicted ΔG values.

From the Additional file 1: Table S1 it is evident that the predicted activation barriers for all the reactions are in good agreement with their corresponding experimental results. A detailed comparison of experimental and predicted activation barriers is evident from Figure 2, which includes also the Artificial Neural Network model results (ANN).

### Nonlinear QSABR model details

The error back-propagation neural network model was developed with 55 training data (55 reactions, see Additional file 1: Table S1). The ANN which was chosen as the best model had input layer with seven neurons, five hidden neurons and one neuron in the output layer. Optimal parameters were chosen according to the lowest RMS errors obtained from Leave-one-out on training data, see Additional file 2: Figure S1, taking into account that the residuals should be randomly distributed around zero. The model was tested by 17 test objects as explained in MLR modeling approach. The predictions for all models and calculations from Tang [13] are compiled in Figure 2.

**Figure 3:**
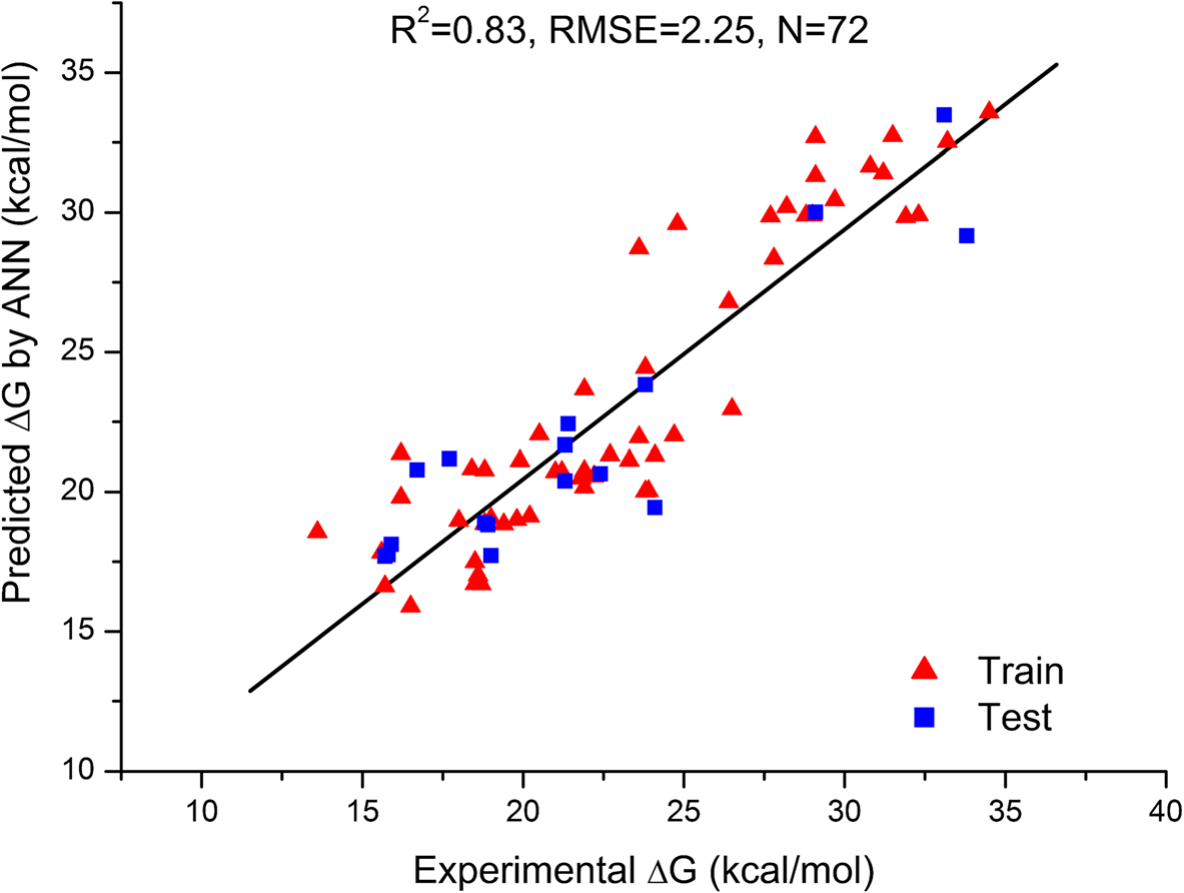
Comparison of experimental activation barriers with those predicted by ANN-model.

BP-ANN with 5 hidden neurons was trained during 4800 epochs with learning rate 0.6 and momentum 0.2. Observed RMS error was 2.22 kcal/mol and 2.33 kcal/mol for the training set objects and testing set objects, respectively. Leave-one-out for the training set objects resulted in RMS error equal to 2.74 kcal/mol. Predictive R_pred_^2^ for test set was 0.83 and Q_Loo_^2^ was 0.76. The regression plot of experimental versus predicted ΔG values by the BP-ANN model is shown in Figure 3.

There is a small difference between RMS errors for ANN and MLR model which is slightly in favour of the developed MLR model. This result implies that the nonlinearity in the relationship between the descriptors (*LUMO*_*d*_, ∆*E*, *ω*_*a*_, (*ω*_*d*_ - *ω*_*a*_), ∆*LUMO*, *SOF*_*d*_, and *DM*_*a*_) and the property (activation barrier) for the studied Diels Alder reactions is not essential and does not represent a problem for QSABR models. When comparing RMS error of predictions (training + test set results) for Tang’s, MLR and ANN model, we observe the values 1.76, 2.14, and 2.25 respectively. We can observe in Figure 2, that in general ΔG values predicted by all three models match well with the experimental ones. While Tang’s results show very good fit for the set of reactions we have used as test set objects, their predictions for the reactions from our training set seem for most of the cases a little below experimental values, especially in the case of reactions 30, 37, 70, 71, and 72 (see Figure 2 and Additional file 1: Table S1 for details). In a closer examination of the prediction errors, there are approximately 84% of values lower than experimental ones in Tang’s calculations, and around 45% in MLR and ANN models. The average residuals of all predictions are -1.28, 0.29 and 0.14 kcal/mol in Tang’s calculation, MLR and ANN, respectively. Although the results are acceptable, they are suggesting a moderate systematic error in Tang’s calculations. Since we are interested in the diene-dienophile pairs which give low activation barrier, the values with the predictions with lowest ΔG values are the most interesting. And as we shall see in the following section, all three models identify the same new reactions as the most promising ones.

### Prediction of activation barriers for new reactions

**Figure 4:**
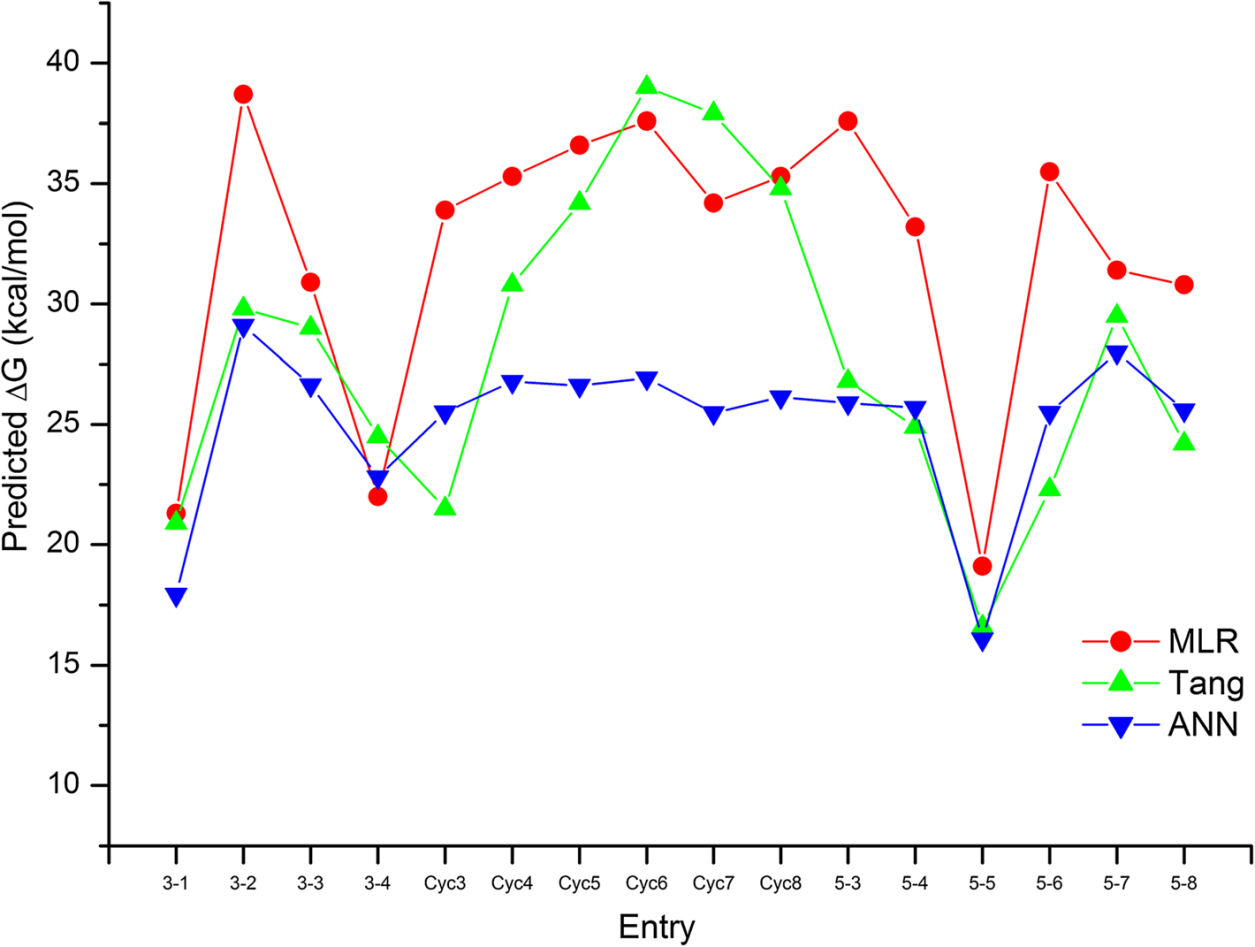
**Predicted activation barriers for new reactions listed in Additional file****3****: Table S2**

The linear and nonlinear models developed were further applied to predict Diels-Alder activation barriers of σ-electron withdrawing group substituted alkenes, cyclic alkenes with consideration of ring strain effect and barriers of Diels-Alder reactions which are promising and nucleophile-tolerant in the living system. These were compared with the activation barriers calculated by Tang et al. as shown in Additional file 3: Table S2 and Figure 4. Structural optimization and descriptor computations were performed for cyclic alkenes (Cyc3 to Cyc8) considering ring strain effect. Prediction of activation barriers of these cyclic alkenes in MLR model follow the trends as obtained by Tang et al. Increasing the angle, barriers increase in the following sequences: cyclopropene (angle = 64.6°; +33.9 kcal/mol) < cyclobutene (angle = 94.4°; +35.3 kcal/mol) < cyclopentene (angle = 112.1°; +36.6 kcal/mol) < Cyclohexene (angle = 123.5°; +37.6 kcal/mol). Cyclohexene produces highest barrier prediction which goes to decreasing for larger homologs such as cycloheptene (+34.2 kcal/mol) and cyclooctene (+35.3 kcal/mol).

The above comparative results between ∆G calculated (by Tang et al. [13]) and predicted (by our MLR and ANN models) indicated that the current theoretical protocol based on the modelling of quantitative structure activation barriers relationship utilizing computed quantum chemical descriptors. MLR model could satisfactorily predict the activation energy barriers of Diels-Alder ligation reactions if compared to Tang’s calculations, except for the entries 3–2, cyc3, 5–3, 5–4 and 5–6 for which deviations from Tang's calculations are higher. Conversely, in the ANN model the four mentioned predictions match well with Tang’s calculations, while the deviations are higher for the reactions with cyclic alkenes as dienophiles (Cyc4 – Cyc8, see Figure 4).

Prediction of activation barrier using theoretical quantum chemical descriptors based protocol is quite easy process and less time consuming. So such model can be applied to predict activation barrier of Diels-Alder ligation reactions before experimental investigation to gain insight for designing of promising Diels-Alder reactions. There is a concern regarding the influence of the solvent in the newly proposed approach. We have observed that the calculated quantum chemical descriptors did not differ significantly for the same reaction performed in different solvents, as for example the reactions 17 and 43 (see Additional file 1: Table S1), although the experimental reaction barriers were significantly different. Consequently, our QSABR model cannot differentiate well these two reactions. On the other hand, we are proposing this fast and efficient new methodology for identification of new click reactions for bio systems, so they are run in water.

**Figure 5:**
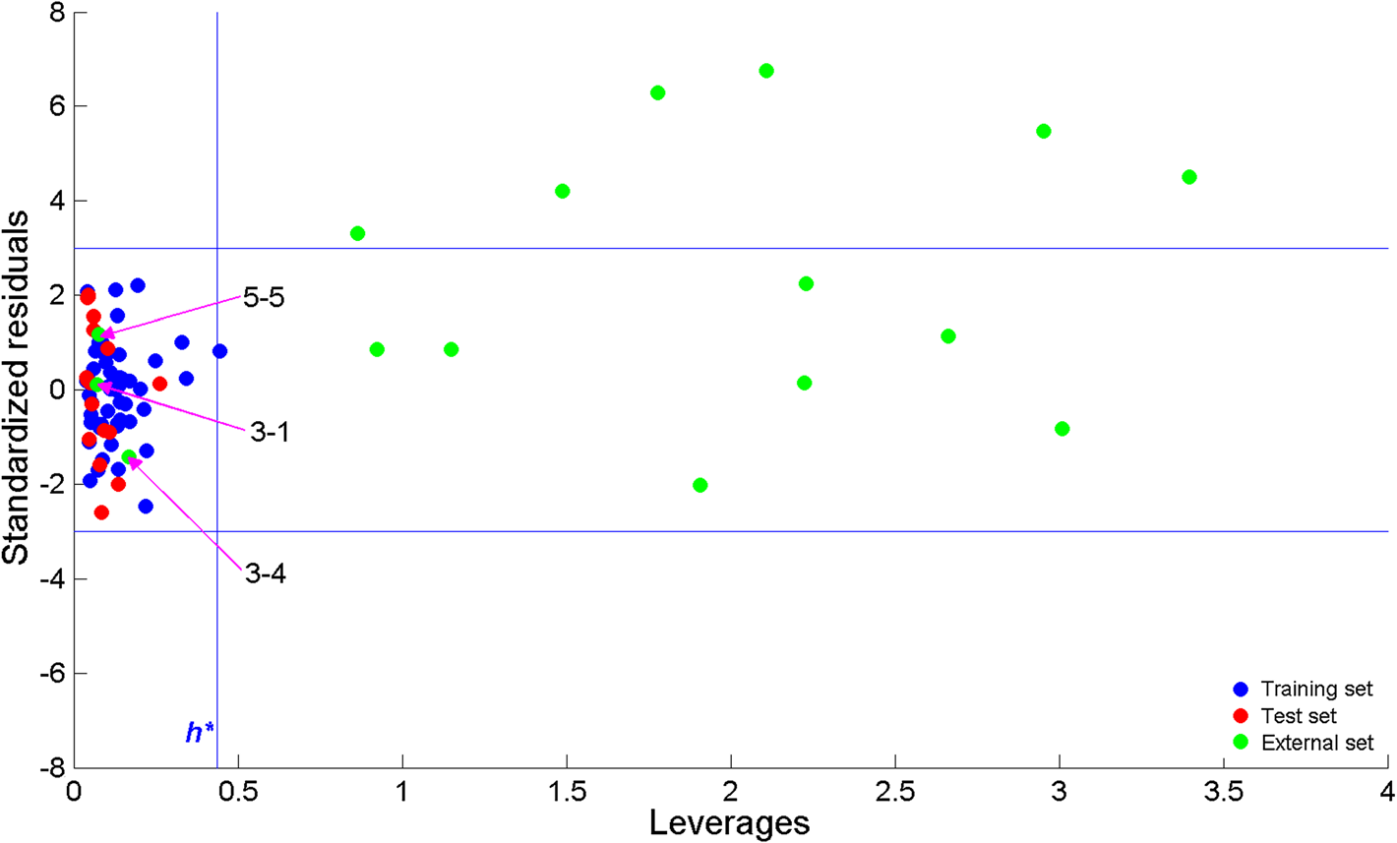
Assessment of the applicability domain for the MLR model.

After the MLR model was built, its applicability domain was assessed for the reactions under the study. The applicability domain was investigated using leverage approach method as described in [61-63]. The results are shown in Figure 5 where blue, red and green dots represent training set, test set and prediction set reactions, respectively. Horizontal blue lines represent cut-off value for standardized residuals and the vertical blue line shows the warning value for leverage values obtained from hat matrix. It can be observed that practically all reactions in the training and test set may be considered as inside the applicability domain, while most of the reactions in the prediction set do not belong to the applicability domain of the MLR model. Only for 3 reactions in the prediction set it can be stated that the MLR model is suitable and reliable predictions have been made. The three reactions are 3–1, 3–4 and 5–5 which have also been found to be reactions with low activation barriers as predicted by the MLR model. Since no experimental values were available for the new reactions, the predictions for the new reactions were compared with Tang’s calculations. Williams plot in Figure 5 shows the importance of applicability domain assessment. From the figure it is visible that approximately half of the applicability domain outliers can be considered also as prediction outliers.

### Experimental

*In silico* experiments have been performed for 72 non-catalyzed Diels-Alder reactions of different dienes and dienophiles with experimentally determined reaction rates obtained from literature [19,39-43]. The activation barriers of 72 reactions calculated by Eyring–Polanyi equation [2] were used as target properties in the QSABR modelling, while the quantum chemical structural descriptors were calculated from structures of standalone reactants, different dienes and dienophiles listed in Additional file 1: Table S1. The structures of all the reactant molecules have been prepared in the computer readable form by Chem3D Ultra [64]. The geometries of reactants were fully optimized at the HF/6-31G(d) level to obtain most stable conformation. Quantum chemical descriptors were calculated utilizing Gaussian 09 software [44]. For all quantum chemical calculations, the solvents specified in the study by Tang et al. [13] have been considered. Solvents are introduced by their dielectric constants (*ϵ*) under the Polarizable Continuum Model (PCM) [65]. The solvents used for corresponding reactions were: Benzene (*ϵ* = 2.2706); Acetonitrile (*ϵ* = 35.688); Water (*ϵ* = 78.3553); 1,4-Dioxane (*ϵ* = 2.2099).

## Conclusions

Quantitative structure-activation barrier relationship studies have been performed utilizing computed quantum chemical descriptors solely calculated from a number of different dienes and dienophiles constituting 72 non-catalyzed Diels-Alder reactions. Stepwise algorithm has been applied for variable selection and the QSABR models have been developed by MLR method. QSABR model reveals that quantum chemical descriptors produce significant impact on the activation barriers for these reaction data. Quantum chemical descriptors such as LUMO level of the diene, difference between softness and difference between the HOMO levels of the dienes and dienophiles, difference between LUMO energies of the dienes and dienophiles, electrophilicity of the dienophiles and dienes and its differences, electronegativity of the dienes, dipole moment of the dienophile, softness of the dienes and orbital interaction energy difference (∆*E*) of dienes and dienophiles (as depicted in Eqs. ([6]) and ([10])) have crucial influences on the activation barrier of the Diels-Alder ligations. Reliability of the training QSABR model was confirmed by statistical validation and thus, the developed QSABR model has been used to predict the Diels-Alder activation barriers of a new set of reactions between 1,3-butadiene and σ-electron withdrawing group substituted alkenes and cyclic alkenes designed and calculated by Tang et al. [13].

It is evident from comparative study of calculated and predicted activation barriers (reported in Additional file 3: Table S2) that our theoretical protocol using computed molecular descriptors of reactants alone can provide a good quality predictive model for the Diels-Alder ligation considered in the present investigation. The aim of this work was to show how the molecular orbital theory provides the descriptors of molecular structure that are sufficient to find reasonable correlation with the reaction barriers. Alternatively, the “atoms in molecules” analysis [64] would provide useful information (descriptors) on electronic structure of the molecules. However, regarding its inherent theoretical framework it decouples from molecular orbital concept of bonding and can thus not supplement our present approach. Nevertheless, the critical points data [64,65] might serve as an alternative source of the stand-alone descriptors which could potentially be successful in predicting chemical reactivity.

With the calculation method described in our work, which considers reactant molecules separately, no problems with convergence were observed. It is also less time consuming than the computationally demanding approach based on the transition state theory; yet, the reliable predictions of the activation barriers can be obtained as demonstrated on a large set of Diels-Alder reactions. Since the proposed model for prediction of activation barriers is based on relatively simple calculations of HOMO and LUMO energies, it can become widely used by the experimentalists in organic chemistry labs as a cheap theoretical method to design new promising Diels-Alder reactions.

## Methods

### Computational section

Molecular orbital theory was applied to calculate quantum-chemical descriptors (all based on the HOMO, LUMO energies and dipole moments). The calculations were performed on high efficient workstation having an Intel(R) Xeon(R) CPU E5620 (12 GB RAM) with the Windows 64-bit operating system. Chem3D Ultra [66] was used to input chemical structures; Gaussian 09 [44] was applied for geometry optimization and quantum chemical calculations; dielectric constants of corresponding solvents were considered in the Polarizable Continuum Model (PCM) [67].

### Data processing and model development by Stepwise-MLR

Multivariate linear regression (MLR) analysis, one of the oldest data reduction methodologies, continues to be widely used in Quantitative Structure Activity/Property/Reactivity (QSAR, QSPR, QSRR) studies [10,68,69]. Selection of variables having significant influence on the reactivity is a key step in QSPR modelling of these reactants to eliminate the problems like chance correlations and multicollinearity. However, because of the co-linearity problem in MLR analysis, we removed the collinear descriptors. Utilizing every available descriptor that may produce a predictive model with a good correlation coefficient, makes the models difficult to interpret and do not stand up to external validation. An integral aspect of model development is to build the model with a small but appropriate set of descriptors with a view to interpret the relationships. This process forms the basis of a technique known as feature selection or variable selection. Among several search algorithms, stepwise forward-backward based feature selection coupled with MLR is the most popular method for building quantitative models and can explain the situation more effectively [60].

Statistical analyses have been carried out by using Minitab software [70]. One has to choose the values of the F statistic for the partial F tests that will determine if a variable is to enter or be removed from the model; F = 0.25 has been chosen as the threshold for inclusion and exclusion of variables.

Initially, the stepwise method searches for an independent variable which correlates the most with the dependent variable. This variable is used as the first variable in the model. Next, from the remaining set of unused independent variables a new variable is selected so that the most of the response’s variance is explained while keeping high values of F-test and correlation coefficient of the model. New variables are being added to the model until no significant improvement of the model can be made. In each consecutive step of adding a variable, the current model is also tested if the quality of the model can be improved by removing any previously selected variable, which is then discarded. Leave-one-out cross-validation was used to evaluate the stability and prediction ability of the generated models in each step.

### Nonlinear modelling by neural networks

Artificial neural network based on back propagation of errors algorithm was applied as a nonlinear modelling method. The same training data were used as for MLR modelling. Back-propagation artificial neural network model (BP-ANN model) is usually presented as a “black box” which consists of input layer, hidden layer and output layer of neurons. Here only a brief description is given to clarify this “black box”, while a more detailed description can be found in literature [71]. The input layer receives input signals that consist of independent variables presenting the inherent properties of the data set objects. The input signals are usually read from a text file; in this study the same descriptors were used as for MLR modelling. The input signals are transferred from the inactive input layer to the active hidden and output layers, in which the signals are transformed through the activation function. The neurons in the hidden and output layers are iteratively modified according to the input signals which are loaded repeatedly to the network until it has been sufficiently trained, which means that the output signals for each input match with the target values (desired properties of each object). The trained network provides us with predictions of one or more predefined properties that are located in the output layer.

Each active neuron transforms input signals using activation function which is usually sigmoidal function. For the first active layer of neurons (hidden layer), the neuron’s output signal is calculated as given in Eq. (11), where *Net* is defined by Eq. ([12]) as a sum of descriptors values *x*_*i*_, multiplied by the corresponding weights *w*_*i*_, *n* being the number of inputs (descriptors). Bias, usually 1, is added to the sum thus *n* + 1 terms.

(11) $$\mathrm{out}=\frac{1}{1+e^{-\mathrm{Net}}}$$

(12) $$\mathrm{Net}=\sum_{i=1}^{n+1}w_{i}x_{i}$$

For the neurons in the subsequent layers, the inputs (*x*_*i*_ values) represent the outputs (*out* values) of the neurons from the preceding layer, while *w*_*i*_ values refer to the neurons of the selected layer. As mentioned above, the network needs to be trained with known input-target pairs of data. That means that appropriate values for all weights in the network should be found. Therefore a sufficiently large set of objects with known descriptor and target values is needed to tune the weights so that the outputs in the layer are reasonably close to the target values. Using the training set objects, BP-ANN is trained by back propagation of errors. The correction of the weights is usually made immediately after each input (single object) and can be summarized using Eq.([13]).

(13) $$\Delta w_{\mathrm{jk}}^{l}=\phi \delta_{j}^{l}\mathrm{ou}t_{k}^{l-1}+\gamma \Delta w_{\mathrm{jk}}^{l\left(\mathrm{previous}\right)}$$

Eq. ([13]) demonstrates how a correction of a weight is calculated for the neuron *j* in the current layer *l* for the input received from the neuron *k* in the preceding layer *l*-1. The correction is made using two terms. The first term shows that correction considers the error produced in the current neuron (*δ*^*l*^_*j*_) and the output from the neuron in the layer above (*out*_*k*_^*l*-1^) which is multiplied by the learning rate (*φ*). The second term is used to reduce oscillations during the learning process and to overcome local minima. It takes the most recent correction of the weight (∆*w*_*jk*_^*l*(*previous*)^) multiplied by a momentum (γ). When each input object has been used once to correct all weights in ANN, the learning procedure has passed one *epoch*. The learning rate and momentum are the two most important parameters, besides the network architecture, the number of hidden layers and the number of neurons in the hidden layers, which must be chosen before the training. Usually the parameters *φ* and *γ* are decreasing with time during the learning procedure [71].

For the purpose of this study, BP-ANN had one hidden layer with one to five neurons, subjected further to a standard procedure of optimization of network parameters.

### Applicability domain assessment

As proposed in the report of the European Centre for the Validation of Alternative Methods [62], the applicability domain of a QSAR model is the response and chemical structure space in which the model makes predictions with a given reliability. Or in other words, as written by Eriksson et al. [63], applicability domain of QSAR model is the range within which it tolerates a new molecule. Thus, if new molecules, or as in our case Diels-Alder reactions, represented by chemical descriptors fall outside applicability domain of the model, the predictions of the model become unreliable.

The applicability domain of the MLR model was assessed using leverage approach method, where the leverage of object *i* can be calculated as *h*_*i*_ = *x*_*i*_^*T*^(*X*^*T*^*X*)^-1^ where *x*_*i*_ is a vector describing object *i* and *X* is a matrix composed of the objects used in the training. The warning leverage, h*, is defined as 3(*n*_*d*_ + 1)/*n*_*o*_, where *n*_*d*_ and *n*_*o*_ are number of descriptors and number of objects used in the training set [62]. If the object’s leverage exceeds the warning level value, the prediction for that objects is considered as unreliable, i.e. the object is outside the model’s applicability domain.

## Abbreviations

ANN: Artificial neural network; BP-ANN: Back-propagation artificial neural network; HOMO: Highest occupied molecular orbital; LUMO: Lowest unoccupied molecular orbital; MLR: Multiple linear regression; PRESS: Predicted residual error sum of squares; QLoo: Leave-one-out cross-validated regression coefficient; QSABR: Quantitative structure-activation barrier relationship; QSAR: Quantitative structure-activity relationship; QSPR: Quantitative structure–property relationship; QSRR: Quantitative structure-reactivity relationship; R: Regression coefficient; SE: Standard error.

## Competing interests

The authors declare that they have no competing interests.

## Authors’ contributions

SN calculated quantum chemical structural parameters for all reactants in 72 Diels-Alder reactions and developed the initial MLR model. AM selected the dataset from literature and advised the chemical optimization steps in QC calculations. VD developed ANN models, assessed applicability domain of MLR model. FM assisted in optimization of QM calculations and interpretation of the results. MN initiated the research and analysed the results. All authors contributed to the preparation of the manuscript. All authors read and approved the final manuscript.

## Supplementary Material

Additional file 1: Table S1 Structures of diene and dienophiles along with activation barrier (∆G) data. Click here for file

Additional file 2: Figure S1 RMS errors and correlation coefficients of different models and distribution of residuals for the selected model. According to low RMS error obtained with Leave-one-out validation method (a), good correlation coefficient (b) and acceptable distribution of residuals in normal probability plot (c) model 30 was chosen as optimal. Click here for file

Additional file 3: Table S2 Predicted activation barriers for new reactions. Click here for file
