# Supplementary material for: Quantitative structure-activation barrier relationship modeling for Diels-Alder ligations utilizing quantum chemical structural descriptors
Source: Chem Cent J. 2013 Oct 30;7:171. doi: 10.1186/1752-153X-7-171 (PMC4176756; doi:10.1186/1752-153X-7-171)
Supplement: Additional file 1: Table S1 — Structures of diene and dienophiles along with activation barrier (∆G) data. [file 1752-153X-7-171-S1.doc]

## Table 1 - Structures of diene and dienophiles along with activation barrier (∆G) data.

| Entry | Diene  (d) | Dienophile  (a) | Experimental ^#^  (∆G) | Predicted  (∆G)  (MLR) | Predicted  (∆G)  (ANN) |
| --- | --- | --- | --- | --- | --- |
| 1 |  |  | 26.5 | 28.3 | 23.0 |
| 2 |  |  | 24.7 | 24.4 | 22.0 |
| 3 |  |  | 23.6 | 24.0 | 22.0 |
| 4 |  |  | 23.3 | 21.4 | 21.1 |
| 5^*^ |  |  | 22.4 | 20.6 | 20.6 |
| 6 |  |  | 22.2 | 21.1 | 20.6 |
| 7 |  |  | 21.8 | 21.0 | 20.5 |
| 8 |  |  | 21.9 | 20.7 | 20.2 |
| 9^*^ |  |  | 21.3 | 21.0 | 20.4 |
| 10 |  |  | 21.0 | 21.0 | 20.7 |
| 11 |  |  | 22.7 | 19.7 | 21.3 |
| 12^*^ |  |  | 16.7 | 19.9 | 20.8 |
| 13 |  |  | 21.9 | 21.7 | 20.8 |
| 14 |  |  | 23.8 | 20.8 | 20.0 |
| 15^*^ |  |  | 24.1 | 20.6 | 19.4 |
| 16 |  |  | 16.5 | 17.2 | 15.9 |
| 17^*^ |  |  | 15.9 | 19.9 | 18.1 |
| 18 |  |  | 15.6 | 19.9 | 17.8 |
| 19^*^ |  |  | 15.8 | 19.8 | 17.7 |
| 20^*^ |  |  | 15.7 | 19.8 | 17.7 |
| 21 |  |  | 15.7 | 19.0 | 16.6 |
| 22 |  |  | 18.5 | 20.7 | 17.5 |
| 23 |  |  | 18.6 | 20.7 | 17.0 |
| 24 |  |  | 18.6 | 20.6 | 16.9 |
| 25 |  |  | 18.5 | 20.6 | 16.7 |
| 26 |  |  | 19.0 | 19.6 | 19.0 |
| 27^*^ |  |  | 18.8 | 19.5 | 18.9 |
| 28^*^ |  |  | 18.9 | 19.5 | 18.8 |
| 29 |  |  | 18.8 | 19.544 | 18.9 |
| 30 |  |  | 13.6 | 18.1 | 18.6 |
| 31 |  |  | 16.2 | 18.0 | 19.8 |
| 32^*^ |  |  | 21.3 | 18.6 | 21.7 |
| 33 |  |  | 21.2 | 19.9 | 20.7 |
| 34 |  |  | 23.8 | 24.1 | 24.4 |
| 35^*^ |  |  | 17.7 | 20.4 | 21.2 |
| 36^*^ |  |  | 21.4 | 20.1 | 22.4 |
| 37 |  |  | 16.2 | 18.0 | 21.4 |
| 38 |  |  | 18.4 | 20.6 | 20.8 |
| 39 |  |  | 18.7 | 18.5 | 16.7 |
| 40 |  |  | 19.9 | 17.3 | 21.1 |
| 41 |  |  | 21.9 | 22.2 | 23.7 |
| 42 |  |  | 18.8 | 19.9 | 20.8 |
| 43^*^ |  |  | 19.0 | 19.5 | 17.7 |
| 44^*^ |  |  | 23.8 | 24.3 | 23.8 |
| 45 |  |  | 24.1 | 20.7 | 21.3 |
| 46 |  |  | 20.5 | 19.4 | 22.1 |
| 47 |  |  | 23.6 | 27.9 | 28.7 |
| 48 |  |  | 24.8 | 26.5 | 29.6 |
| 49 |  |  | 26.4 | 27.8 | 26.8 |
| 50 |  |  | 27.7 | 28.1 | 29.9 |
| 51 |  |  | 27.8 | 28.0 | 28.4 |
| 52 |  |  | 28.2 | 27.6 | 30.2 |
| 53 |  |  | 28.8 | 27.7 | 29.9 |
| 54 |  |  | 29.0 | 27.8 | 29.9 |
| 55^*^ |  |  | 29.1 | 31.0 | 30.0 |
| 56 |  |  | 29.1 | 31.3 | 31.3 |
| 57 |  |  | 29.1 | 29.5 | 32.7 |
| 58 |  |  | 29.7 | 31.5 | 30.4 |
| 59 |  |  | 30.8 | 32.1 | 31.7 |
| 60 |  |  | 31.2 | 32.2 | 31.4 |
| 61 |  |  | 31.5 | 32.1 | 32.7 |
| 62 |  |  | 31.9 | 31.4 | 29.8 |
| 63 |  |  | 32.3 | 33.0 | 29.9 |
| 64^*^ |  |  | 33.1 | 28.4 | 33.5 |
| 65 |  |  | 33.2 | 28.8 | 32.5 |
| 66^*^ |  |  | 33.8 | 32.4 | 29.2 |
| 67 |  |  | 34.5 | 32.6 | 33.6 |
| 68 |  |  | 23.9 | 21.7 | 20.0 |
| 69 |  |  | 20.2 | 19.1 | 19.1 |
| 70 |  |  | 19.8 | 18.9 | 19.0 |
| 71 |  |  | 19.4 | 18.4 | 18.8 |
| 72 |  |  | 18.0 | 18.7 | 19.0 |
|  |  |  |  |  |  |
| ^*^ Test reactions; Units: ∆G (exp. & pred.) =kcal/mol  ^#^ Exp. (∆G) are obtained by Eyring–Polanyi equation [2] given as:  *k*=(*k_B_T*/*h*)e^-Δ^*^G^*^#/(^*^RT^*)  *k* = reaction rate constant, *T*= absolute temperature, *R*= gas constant, *k_B_*= Boltzmann constant (1.38 x10^-23^ JK^-1^), *һ*= Planck's constant (6.6261 x10^-34^ Js) | | | | | |
