# Supplementary material for: Quantitative structure-activation barrier relationship modeling for Diels-Alder ligations utilizing quantum chemical structural descriptors
Source: Chem Cent J. 2013 Oct 30;7:171. doi: 10.1186/1752-153X-7-171 (PMC4176756; doi:10.1186/1752-153X-7-171)
Supplement: Additional file 2: Figure S1 — RMS errors and correlation coefficients of different models and distribution of residuals for the selected model. According to low RMS error obtained with Leave-one-out validation method (a), good correlation coefficient (b) and acceptable distribution of residuals in normal probability plot (c) model 30 was chosen as optimal. [file 1752-153X-7-171-S2.doc]

a)

b)

c)

**Figure S1: RMS errors and correlation coefficients of different models and distribution of residuals for the selected model.**  According to low RMS error obtained with Leave-one-out validation method (a), good correlation coefficient (b) and acceptable distribution of residuals in normal probability plot (c) model 30 was chosen as optimal.
