# Supplementary material for: Quantitative structure-activation barrier relationship modeling for Diels-Alder ligations utilizing quantum chemical structural descriptors
Source: Chem Cent J. 2013 Oct 30;7:171. doi: 10.1186/1752-153X-7-171 (PMC4176756; doi:10.1186/1752-153X-7-171)
Supplement: Additional file 3: Table S2 — Predicted activation barriers for new reactions. [file 1752-153X-7-171-S3.doc]

## Table 3 - Predicted activation barriers for new reactions.

| Entry | Diene | Dienophile | Calculated (∆G)  (by Tang et al. [13]) | Predicted (∆G) | |
| --- | --- | --- | --- | --- | --- |
|  |  |  |  | Using Eq.(10) | By ANN model |
| 3-1 |  |  | 20.9 | 21.3 | 17.9 |
| 3-2 |  |  | 29.8 | 38.7 | 29.1 |
| 3-3 |  |  | 29.0 | 30.9 | 26.6 |
| 3-4 |  |  | 24.5 | 22.0 | 22.8 |
| Cyc3 |  | (Angle= 64.6°) | 21.5 | 33.9 | 25.5 |
| Cyc4 |  | (Angle= 94.4°) | 30.8 | 35.3 | 26.8 |
| Cyc5 |  | (Angle=112.1°) | 34.2 | 36.6 | 26.6 |
| Cyc6 |  | (Angle=123.5°) | 39.0 | 37.6 | 26.9 |
| Cyc7 |  | (Angle= 127.9°) | 37.9 | 34.2 | 25.5 |
| Cyc8 |  | (Angle= 121.5°) | 34.8 | 35.3 | 26.1 |
| 5-3 |  |  | 26.8 | 37.6 | 25.9 |
| 5-4 |  |  | 24.9 | 33.2 | 25.7 |
| 5-5 |  |  | 16.6 | 19.1 | 16.1 |
| 5-6 |  |  | 22.3 | 35.5 | 25.5 |
| 5-7 |  |  | 29.5 | 31.4 | 28.0 |
| 5-8 |  |  | 24.2 | 30.8 | 25.6 |
| Unit of (∆G)=kcal/mol | | | | | |
